# Outcomes of Gastroparesis in Hospitalized Patients With Generalized Anxiety Disorder

**DOI:** 10.7759/cureus.35832

**Published:** 2023-03-06

**Authors:** Anna G Mathew, Alexander J Kaye, Shivani J Patel, Sarah R Meyers, Pooja Saiganesh, Weizheng Wang

**Affiliations:** 1 Internal Medicine, Rutgers University New Jersey Medical School, Newark, USA; 2 Psychiatry, Rutgers Robert Wood Johnson Medical School, Piscataway, USA; 3 Gastroenterology and Hepatology, Rutgers University New Jersey Medical School, Newark, USA

**Keywords:** vomiting, nausea, diabetes mellitus, gastroparesis, generalized anxiety disorder, acute renal failure

## Abstract

Background

Gastroparesis is a common gastrointestinal pathology that has been increasing in prevalence and represents a significant cost to the United States healthcare system. Gastroparesis is associated with psychological dysfunction, including generalized anxiety disorder (GAD). GAD is known to be a prevalent and chronic manifestation of anxiety, which has been increasing in prevalence since the year 2020. Despite the association between gastroparesis and GAD, there has been limited research on the possible impact GAD may have on the morbidity and mortality of patients hospitalized for gastroparesis, which is further evaluated in this study.

Methods

Using the Nationwide Inpatient Sample from the year 2014, a retrospective study was conducted to assess the outcomes of hospitalized gastroparesis patients with and without a history of GAD. In this study, the analyzed outcomes included acute kidney injury (AKI), acute respiratory failure, sepsis, acute deep vein thrombosis, myocardial infarction, intestinal obstruction, and inpatient mortality. To assess whether GAD is an independent risk factor for the outcomes, a multivariate logistic regression analysis was used.

Results

There were 22,150 patients with gastroparesis assessed in this study; GAD was found to be a comorbid diagnosis in 4,196 of those patients. In the GAD cohort, there was an elevated risk for AKI (adjusted odds ratio 1.24, p < 0.001). The adjusted odds ratios for acute respiratory failure, sepsis, acute deep vein thrombosis, myocardial infarction, intestinal obstruction, and inpatient mortality did not meet the threshold for statistical significance.

Conclusion

In hospitalized gastroparesis patients, GAD is a risk factor for AKI. This finding may be attributed to prerenal azotemia due to an increased risk of nausea and vomiting associated with GAD, as well as the medications used to treat GAD such as escitalopram and duloxetine. In addition, the dual inflammatory states caused by the co-existence of both GAD and gastroparesis may also have a role in increasing the risk for AKI. The results of this study may become increasingly relevant given the increasing prevalence of GAD.

## Introduction

Gastroparesis is a well-known and debilitating gastrointestinal pathology. This condition has become increasingly common in recent years with the prevalence rising to 267.7 per 100,000 United States (US) adults in 2018 [1,2]. Subsequently, gastroparesis represents a significant expense to the healthcare system; in 2017, it cost the US healthcare system an estimated 550 million dollars [3].  

Gastroparesis is characterized by the delayed emptying of solid food from the stomach, in the absence of mechanical obstruction, commonly leading to nausea, bloating, loss of appetite, severe abdominal pain, and gastroesophageal reflux disease [4]. The pathophysiology of gastroparesis is still under investigation, but previous studies have established the involvement of oxidative stress and inflammation in pyloric dysfunction [4]. Treatment of gastroparesis can include dietary interventions such as portion reduction, pharmacological agents such as prokinetics and antiemetics, as well as procedural interventions such as gastric electrical stimulation and pyloromyotomy [4]. Diabetes mellitus is the most frequent etiology of gastroparesis, but other known causes include surgery and medications [1,5].  

Gastroparesis has been previously associated with psychological dysfunction, including generalized anxiety disorder (GAD) [6,7]. As one of the most common psychiatric disorders, GAD has a lifetime prevalence of 3.7% across adults aged 18-99 years [8]. This disorder is marked by at least six months of persistent and excessive anxiety and can include physical symptoms such as muscle tension, insomnia, and fatigue as outlined in the Diagnostic and Statistical Manual of Mental Disorders, fifth edition (DSM-5) [9]. Notably, the prevalence of GAD has been increasing since the year 2020 [10]. Treatment of GAD can often incorporate selective serotonin reuptake inhibitors (SSRIs) or serotonin and norepinephrine reuptake inhibitors (SNRIs), as well as cognitive behavioral therapy [11].  

Although gastroparesis has been previously linked to GAD, there has been limited data on the impact of GAD on the morbidity and mortality of hospitalized gastroparesis patients. This research evaluates the hospital-based outcomes of patients who are admitted for gastroparesis with a history of GAD.

This research was displayed as a poster at the American College of Gastroenterology conference on October 24, 2022. 

## Materials and methods

This retrospective cohort study was performed to investigate all adult patients who were hospitalized in the year 2014 due to gastroparesis. Institutional review board approval was not required for this study as no patient-level data were used. The data in this research were collected from the Nationwide Inpatient Sample (NIS), a database created for the Healthcare Cost and Utilization Project (HCUP), which is sponsored by the Agency for Healthcare Research and Quality [12]. The NIS database is recognized as the largest all-payer database for hospitalized patients in the United States [12]. The diagnoses evaluated within the study were identified using International Classification of Diseases, Ninth Edition Revision, Clinical Modification (ICD-9) codes from within the NIS database. Patients hospitalized for gastroparesis were identified and subsequently distributed between two cohorts, one cohort with comorbid GAD and the other cohort without comorbid GAD. Between the cohorts, hospitalization information as well as demographic data including hospitalization cost, length of stay, race, sex, and age were obtained and then compared. The Charlson comorbidity index, a tool that can be employed to adjust for multiple confounding variables, was subsequently compared between the group with GAD and the group without GAD [13].  

Statistical Package for the Social Sciences (SPSS) version 28.0 (IBM corporation, Armonk, NY) was utilized for the required statistical analyses. The clinical outcomes evaluated included acute kidney injury (AKI), acute respiratory failure, sepsis, acute deep vein thrombosis, myocardial infarction, intestinal obstruction, and inpatient mortality. Chi-squared tests as well as independent t-tests were utilized to compare proportions and means, respectively, for the demographic and clinical outcomes data of the groups with and without GAD.  

Statistical analyses were two-tailed, utilizing a p-value of below 0.05 to define statistically significant data. Continuous variables were reported with means and standard deviations (SDs), and categorical variables were reported using numbers (N) and percentages (%). A multivariate logistic regression analysis was conducted to explore whether GAD is a risk factor for the outcomes in this study, even after Charlson comorbidity index, race, sex, and age were adjusted for. The familywise error rate for the analyses was not controlled for due to the not well-established advantage of utilizing this technique in addition to the elevated risk for a type 2 statistical error that is related to the correction [14]. 

## Results

For this study, 22,150 adult patients hospitalized with gastroparesis during the year 2014 were identified. These cases were divided into two cohorts, those with a history of GAD (18.9%, n = 4,196) and those without GAD (81.1%, n = 17,954).   

Several demographics and hospitalization characteristics significantly varied between the two cohorts as seen in Table 1. Gastroparesis patients with comorbid GAD had on average a lower age (46.75 years vs. 48.09 years, p < 0.001), were more likely to be women (79.2% vs. 66.1%, p < 0.001), and were composed of a greater proportion of Caucasian patients (71.8% vs. 55.9%, p < 0.001). In addition, the GAD cohort had longer hospitalizations (4.68 days vs. 4.50 days, p = 0.024), as well as a smaller Charlson comorbidity index (2.25 vs. 2.58, p < 0.001). There was no statistically significant difference in total hospital charges ($34,888 vs. $34,601, p = 0.696).  

**Table 1:** Demographics, characteristics, length of stay, total hospital charge, and Charlson comorbidity index among gastroparesis patients with and without a history of generalized anxiety disorder SD, standard deviation.

	With generalized anxiety disorder	Without generalized anxiety disorder	p-Value
N = 22,150	N = 4,196	N = 17,954	
Patient age, mean (SD)	46.75 (16.09)	48.09 (17.53)	<0.001
Sex, N (%)			<0.001
Female	3,324 (79.2%)	11,867 (66.1%)	
Male	871 (20.8%)	6,085 (33.9%)	
Race, N (%)			<0.001
White	2,883 (71.8%)	9,606 (55.9%)	
Black	651 (16.2%)	5,009 (29.1%)	
Hispanic	368 (9.2%)	1,908 (11.1%)	
Asian or Pacific Islander	22 (0.5%)	198 (1.2%)	
Native American	33 (0.8%)	125 (0.7%)	
Other	61 (1.5%)	349 (2.0%)	
Length of stay, in days (SD)	4.68 (5.93)	4.50 (4.50)	0.024
Total hospital charges, in $ (SD)	34,888 (42404.01)	34,601 (42800.40)	0.696
Charlson comorbidity index (SD)	2.25 (2.16)	2.58 (2.31)	<0.001

As observed in Table 2, unadjusted clinical outcomes of patients admitted for gastroparesis significantly varied between the GAD and non-GAD cohorts. Notably, patients with GAD had a decreased likelihood of AKI (10.6% vs. 14.4%, p < 0.001). However, no statistically significant difference in acute respiratory failure (1.2% vs. 1.2%, p = 0.927), sepsis (2.9% vs. 2.9%, p = 0.796), acute deep vein thrombosis (0.3% vs. 0.4%, p = 0.171), myocardial infarction (1.0% vs. 1.0%, p = 0.951), intestinal obstruction (1.1% vs. 1.1%, p = 0.775), and inpatient mortality (0.3% vs. 0.3%, p = 0.984) was observed between the two cohorts.   

**Table 2:** Unadjusted clinical outcomes among gastroparesis patients with and without a history of comorbid generalized anxiety disorder

Outcomes	With generalized anxiety disorder	Without generalized anxiety disorder	p-Value
Acute kidney injury	444 (10.6%)	2,581 (14.4%)	<0.001
Acute respiratory failure	50 (1.2%)	217 (1.2%)	0.927
Sepsis	123 (2.9%)	513 (2.9%)	0.796
Acute deep vein thrombosis	11 (0.3%)	73 (0.4%)	0.171
Myocardial infarction	40 (1.0%)	173 (1.0%)	0.951
Intestinal obstruction	47 (1.1%)	192 (1.1%)	0.775
Inpatient mortality	12 (0.3%)	51 (0.3%)	0.984

To better explore the influence of GAD on this study’s outcomes, adjusted odds ratios (aORs) were calculated, adjusting for differences in age, race, sex, and Charlson comorbidity index as shown in Table 3. Gastroparesis patients with a history of GAD had increased odds of developing AKI (aOR 1.24, 95% confidence interval [CI] 1.11-1.39, p < 0.001). The p-values for the aORs of acute respiratory failure (aOR 0.91, 95% CI 0.66-1.26, p = 0.583), sepsis (aOR 0.92, 95% CI 0.75-1.14, p = 0.455), acute deep vein thrombosis (aOR 1.45, 95% CI 0.76-2.74, p = 0.259), myocardial infarction (aOR 0.81, 95% CI 0.57-1.16, p = 0.256), intestinal obstruction (aOR 1.02, 95% CI 0.73-1.42, p = 0.928), and inpatient mortality (aOR 0.79, 95% CI 0.42-1.50, p = 0.476) were not statistically significant. Both Table 2 and Table 3 discuss similar outcomes; however, the data appear to show conflicting findings. For example, Table 2 shows decreased incidence of AKI in the GAD cohort, yet Table 3 shows an increased OR for AKI in the GAD group. This finding can be attributed to confounding factors, which were adjusted for when calculating the aORs in Table 3.

**Table 3:** Multivariate logistic regression analysis of the outcomes among gastroparesis patients *Adjusted for age, sex, race, and the Charlson comorbidity index.

Outcomes	Adjusted odds ratio*	95% confidence interval	p-Value
Acute kidney injury	1.24	1.11-1.39	<0.001
Acute respiratory failure	0.91	0.66-1.26	0.583
Sepsis	0.92	0.75-1.14	0.455
Acute deep vein thrombosis	1.45	0.76-2.74	0.259
Myocardial infarction	0.81	0.57-1.16	0.256
Intestinal obstruction	1.02	0.73-1.42	0.928
Inpatient mortality	0.79	0.42-1.50	0.476

## Discussion

This is the first study to investigate the impact of comorbid GAD in adult patients hospitalized with gastroparesis. The results showed that hospitalized gastroparesis patients who had a history of GAD were more likely to develop AKI compared to those without GAD. Gastroparesis has been previously associated with renal damage; however, the findings of this study suggest that patients with GAD may be at additional risk for the development of AKI [15].  

Extracellular volume losses through diarrhea, vomiting, and decreased oral intake can lead to prerenal azotemia [16]. Nausea, vomiting, and decreased oral intake from chronic abdominal pain, all of which can lead to volume depletion, can be present in both gastroparesis and GAD [17,18]. It is possible that hospitalized gastroparesis patients with comorbid GAD may experience these symptoms more frequently, thereby increasing the likelihood of volume loss and predisposing patients to renal damage.  

Additionally, patients with GAD may be treated with certain medications that place them at additional risk for AKI. For adults with GAD, SSRIs and SNRIs are the first-line pharmacologic treatment [19]. The most common side effect reported for both SSRIs and SNRIs is nausea [20]. Specifically, the medications with the largest effect size for GAD, escitalopram (SSRI) and duloxetine (SNRI), have been previously linked with higher rates of nausea and vomiting than placebo; patients on escitalopram are over two-fold more likely to develop these side effects while patients on duloxetine are over four-fold more likely to develop nausea and vomiting [21,22]. Therefore, both GAD and its treatments can increase the risk of nausea and vomiting, leading to possible volume loss.  

Finally, systemic inflammation can serve a significant role in the pathophysiology of AKI. Specifically, prior research has established the impact of proinflammatory cytokines, oxidative stress, and intestinal dysbiosis on the chronic inflammatory state of renal failure [23]. Specifically, several markers of inflammation, including interleukin-6 (IL-6) and tumor necrosis factor-α (TNFα), have been particularly implicated in the progression of chronic kidney disease [23]. Motility disorders, including gastroparesis, have been associated with systemic inflammation [24]. The levels of several cytokines, including serum TNFα and IL-6, are elevated in gastroparesis patients [24]. Similarly, GAD has also been associated with an increased pro-inflammatory response, decreased anti-inflammatory response, and altered cytokine balance [25]. Thus, both gastroparesis and GAD have been independently linked to systemic inflammation. Based on our findings, we hypothesize that gastroparesis patients with comorbid GAD may experience a synergistic effect between the two inflammatory states with even more extensive immune dysregulation, increasing the likelihood of developing AKI.  

There are a few notable limitations of this investigation. First, this study was based on the NIS database, which is fully reliant on the accuracy of inputted billing codes by healthcare providers. Thus, inconsistent or incorrect usage of billing codes can misrepresent the prevalence of GAD in gastroparesis patients, as well as affect this study’s outcomes. Also, a history of comorbid GAD may not have been documented in more acute or severe patient presentations, thereby possibly affecting the true prevalence of the condition in our sample. Additionally, the study was unable to specifically account for the use of certain treatments, such as SSRIs or SNRIs, as there is no ICD-9 code available that corresponds to these medications. Similarly, our study was unable to account for possible medication noncompliance, thus additionally affecting clinical outcomes. In addition, the NIS database only includes patients who were hospitalized for gastroparesis, therefore excluding data from gastroparesis patients who were treated exclusively in an outpatient setting. Previous research has indicated that most patients with gastroparesis can be managed as outpatients with only roughly 25% of patients requiring hospitalization [15]. Despite these limitations, one of the notable strengths of this study is the ability to assess demographics and outcomes on a national scale. Another significant strength of this research is the use of a multivariate logistic regression analysis, which adjusted for many confounding variables. 

## Conclusions

In summary, patients hospitalized for gastroparesis with comorbid GAD are at an increased odds of developing AKI. These patients should be monitored for early signs of renal injury. Appropriate monitoring may include more frequent checks of serum creatinine and blood urea nitrogen, minimal use of nephrotoxic medications, as well as close monitoring of patients’ volume status. The relevance and importance of the study results will likely grow given the increasing prevalence of GAD. Further investigation is warranted to evaluate GAD as an independent risk factor for adverse clinical outcomes in gastroparesis patients after controlling for SSRI and SNRI use, given these medications can induce nausea and vomiting. In addition, a future study that investigates outpatient gastroparesis patients with GAD would be helpful in determining whether GAD is a risk factor for AKI outside of the hospital setting. Finally, investigating whether other comorbid diseases that induce systemic inflammation (such as rheumatoid arthritis or systemic lupus erythematosus) are risk factors for AKI in the setting of hospitalized gastroparesis patients would be another possible direction for future research.  

## References

[1] Grover M, Parkman HP, Pasricha PJ (2022). Epidemiology of gastroparesis in the United States. Gastroenterology.

[2] Wadhwa V, Mehta D, Jobanputra Y, Lopez R, Thota PN, Sanaka MR (2017). Healthcare utilization and costs associated with gastroparesis. World J Gastroenterol.

[3] Ye Y, Yin Y, Huh SY, Almansa C, Bennett D, Camilleri M (2022). Epidemiology, etiology, and treatment of gastroparesis: Real-world evidence from a large US national claims database. Gastroenterology.

[4] Camilleri M, Sanders KM (2022). Gastroparesis. Gastroenterology.

[5] Zheng T, Camilleri M (2021). Management of gastroparesis. Gastroenterol Hepatol (N Y).

[6] Teigland T, Iversen MM, Sangnes DA, Dimcevski G, Søfteland E (2018). A longitudinal study on patients with diabetes and symptoms of gastroparesis - associations with impaired quality of life and increased depressive and anxiety symptoms. J Diabetes Complications.

[7] Hasler WL, Parkman HP, Wilson LA (2010). Psychological dysfunction is associated with symptom severity but not disease etiology or degree of gastric retention in patients with gastroparesis. Am J Gastroenterol.

[8] Ruscio AM, Hallion LS, Lim CC (2017). Cross-sectional comparison of the epidemiology of DSM-5 generalized anxiety disorder across the globe. JAMA Psychiatry.

[9] Hobbs MJ, Anderson TM, Slade T, Andrews G (2014). Structure of the DSM-5 generalized anxiety disorder criteria among a large community sample of worriers. J Affect Disord.

[10] Cordaro M, Grigsby TJ, Howard JT, Deason RG, Haskard-Zolnierek K, Howard K (2021). Pandemic-specific factors related to generalized anxiety disorder during the initial COVID-19 protocols in the United States. Issues Ment Health Nurs.

[11] DeMartini J, Patel G, Fancher TL (2019). Generalized anxiety disorder. Ann Intern Med.

[12] (2022). HCUP National Inpatient Sample (NIS): Healthcare Cost and Utilization Project (HCUP). Agency for Healthcare Research and Quality, Rockville, MD. https://www.hcup-us.ahrq.gov/nisoverview.jsp.

[13] Austin SR, Wong YN, Uzzo RG, Beck JR, Egleston BL (2015). Why summary comorbidity measures such as the Charlson comorbidity index and Elixhauser score work. Med Care.

[14] Perneger TV (1998). What's wrong with Bonferroni adjustments. BMJ.

[15] Bielefeldt K (2013). Factors influencing admission and outcomes in gastroparesis. Neurogastroenterol Motil.

[16] Bradshaw C, Zheng Y, Silver SA, Chertow GM, Long J, Anand S (2018). Acute kidney injury due to diarrheal illness requiring hospitalization: Data from the National Inpatient Sample. J Gen Intern Med.

[17] Haug TT, Mykletun A, Dahl AA (2002). Are anxiety and depression related to gastrointestinal symptoms in the general population?. Scand J Gastroenterol.

[18] Parkman HP, Hallinan EK, Hasler WL (2016). Nausea and vomiting in gastroparesis: Similarities and differences in idiopathic and diabetic gastroparesis. Neurogastroenterol Motil.

[19] Garakani A, Murrough JW, Freire RC, Thom RP, Larkin K, Buono FD, Iosifescu DV (2020). Pharmacotherapy of anxiety disorders: Current and emerging treatment options. Front Psychiatry.

[20] Stubbs C, Mattingly L, Crawford SA, Wickersham EA, Brockhaus JL, McCarthy LH (2017). Do SSRIs and SNRIs reduce the frequency and/or severity of hot flashes in menopausal women. J Okla State Med Assoc.

[21] He H, Xiang Y, Gao F (2019). Comparative efficacy and acceptability of first-line drugs for the acute treatment of generalized anxiety disorder in adults: A network meta-analysis. J Psychiatr Res.

[22] Oliva V, Lippi M, Paci R (2021). Gastrointestinal side effects associated with antidepressant treatments in patients with major depressive disorder: A systematic review and meta-analysis. Prog Neuropsychopharmacol Biol Psychiatry.

[23] Rapa SF, Di Iorio BR, Campiglia P, Heidland A, Marzocco S (2019). Inflammation and oxidative stress in chronic kidney disease-potential therapeutic role of minerals, vitamins and plant-derived metabolites. Int J Mol Sci.

[24] Abell TL, Kedar A, Stocker A (2021). Pathophysiology of gastroparesis syndromes includes anatomic and physiologic abnormalities. Dig Dis Sci.

[25] Hou R, Garner M, Holmes C, Osmond C, Teeling J, Lau L, Baldwin DS (2017). Peripheral inflammatory cytokines and immune balance in generalised anxiety disorder: Case-controlled study. Brain Behav Immun.

